# Phosmet: *O,O*-dimethyl *S*-phthalimidomethyl phospho­rodithio­ate

**DOI:** 10.1107/S1600536810029338

**Published:** 2010-07-31

**Authors:** Sanghun Cheon, Hojin Yang, Ki-Min Park, Tae Ho Kim, Jineun Kim

**Affiliations:** a Department of Chemistry and Research Institute of Natural Sciences, Gyeongsang, National University, Jinju 660-701, Republic of Korea

## Abstract

In the title compound, C_11_H_12_NO_4_PS_2_, the dihedral angle between the phthalimidyl ring plane and the PS_2_ plane of the phospho­rodithio­ate group is 60.41 (3)°. In the crystal structure, weak inter­molecular C—H⋯O hydrogen bonds and S⋯S inter­actions [3.3825 (9) Å] contribute to the stabilization of the packing.

## Related literature

For information on the toxicity and insecticidal properties of the title compound, see: Song *et al.* (2009 ▶). For related structures, see: Baughman & Allen (1995 ▶); Rohrbaugh *et al.* (1976 ▶). For the synthesis, see: Sinderhauf & Schwack (2004 ▶).
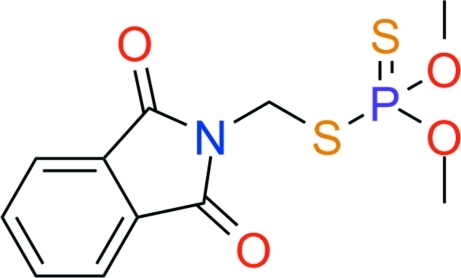


## Experimental

### 

#### Crystal data

C_11_H_12_NO_4_PS_2_
*M* *_r_* = 317.31
Triclinic, 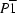

*a* = 8.3428 (18) Å
*b* = 8.6014 (19) Å
*c* = 10.218 (2) Å
α = 85.253 (10)°
β = 81.478 (10)°
γ = 83.961 (9)°
*V* = 719.4 (3) Å^3^
*Z* = 2
Mo *K*α radiation
μ = 0.49 mm^−1^
*T* = 173 K
0.29 × 0.25 × 0.15 mm

#### Data collection

Bruker APEXII CCD diffractometer
Absorption correction: multi-scan (*SADABS*; Sheldrick, 1996 ▶) *T*_min_ = 0.871, *T*_max_ = 0.930
13076 measured reflections
3613 independent reflections
3404 reflections with *I* > 2σ(*I*)
*R*_int_ = 0.025

#### Refinement

*R*[*F* ^2^ > 2σ(*F* ^2^)] = 0.031
*wR*(*F* ^2^) = 0.095
*S* = 1.04
3613 reflections
174 parameters
H-atom parameters constrained
Δρ_max_ = 0.34 e Å^−3^
Δρ_min_ = −0.44 e Å^−3^

### 

Data collection: *APEX2* (Bruker, 2006 ▶); cell refinement: *SAINT* (Bruker, 2006 ▶); data reduction: *SAINT*; program(s) used to solve structure: *SHELXTL* (Sheldrick, 2008 ▶); program(s) used to refine structure: *SHELXTL*; molecular graphics: *SHELXTL* and *DIAMOND* (Brandenburg, 1998 ▶); software used to prepare material for publication: *SHELXTL*.

## Supplementary Material

Crystal structure: contains datablocks global, I. DOI: 10.1107/S1600536810029338/jh2189sup1.cif

Structure factors: contains datablocks I. DOI: 10.1107/S1600536810029338/jh2189Isup2.hkl

Additional supplementary materials:  crystallographic information; 3D view; checkCIF report

## Figures and Tables

**Table 1:** Hydrogen-bond geometry (Å, °)

*D*—H⋯*A*	*D*—H	H⋯*A*	*D*⋯*A*	*D*—H⋯*A*
C2—H2*B*⋯O3^i^	0.98	2.57	3.272 (2)	128
C2—H2*C*⋯O4^ii^	0.98	2.70	3.420 (2)	130

Symmetry codes: (i) 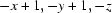; (ii) 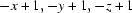.

**Fig. 1.**
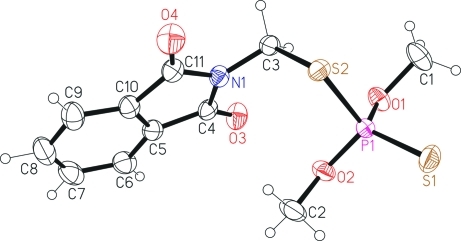
The molecular structure of the title compound, showing displacement ellipsoids drawn at the 50% probability level.

**Fig. 2.**
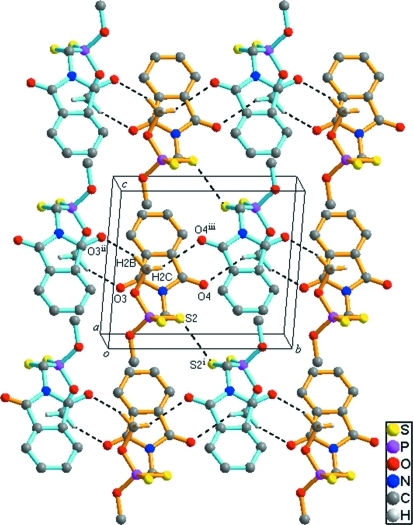
Crystal packing of the title compound with intermolecular C—H···O and S···S interactions shown as dashed lines. H atoms not involved in intermolecular interactions have been omitted for clarity. (Symmetry codes: i) -x + 1, -y, -z + 1; ii) -x + 1, -y + 1, -z; iii) -x + 1, -y + 1, -z + 1)

## supplementary crystallographic information

### Comment

Phosmet (systematic name: *O,O*-dimethyl *S*-phthalimidomethyl phosphorodithioate), is a well known organothiophosphate acaricides and isoindole organothiophosphate insecticides used on plants and animals (Song *et al.*, 2009). However, it's crystal structure has not been reported yet.

In the title compound (Scheme 1, Fig.1), the dihedral angle between the phthalimidyl ring plane and the S1/P1/S2 plane of phosphorodithioate group is 60.41 (3)°. All bond lengths and bond angles of phosphorodithioate group are are comparable to those observed in similar structures (Baughman & Allen, 1995; Rohrbaugh *et al.*, 1976).

In the crystal structure, as shown in Fig. 2, weak C—H···O hydrogen bonds are observed [C2—H2B···O3; H2B···O3 = 2.57 Å; C2—H2B···O3 = 128°; C2···O3 = 3.272 (2) Å; -*x* + 1, -*y* + 1, -*z* and C2—H2C···O4; H2C···O4 = 2.70 Å; C2—H2C···O4 = 130°; C2···O4 = 3.420 (2) Å; -*x* + 1, -*y* + 1, -*z* + 1]. Weak intermolecular S···S interactions with 3.3825 (9) Å also exist. These intermolecular interactions may be contribute to the stabilization of the packing.

### Experimental

The title compound was purchased from the Dr. Ehrenstorfer GmbH Company. Slow evaporation of a CH_2_Cl_2_ solution gave single crystals suitable for X-ray analysis.

### Refinement

All H-atoms were positioned geometrically and refined using a riding model with d(C—H) = 0.95 Å, *U*_iso_ = 1.2*U*_eq_(C) for aromatic and 0.98 Å, *U*_iso_ = 1.5*U*_eq_(C) for the d(C—H) = 0.98 Å, *U*_iso_ = 1.5*U*_eq_(C) for CH_3_ groups.

### Crystal data

**Table:**

C_11_H_12_NO_4_PS_2_	*Z* = 2
*M**_r_* = 317.31	*F*(000) = 328
Triclinic, *P*1	*D*_x_ = 1.465 Mg m^−^^3^
Hall symbol: -P 1	Mo *K*α radiation, λ = 0.71073 Å
*a* = 8.3428 (18) Å	Cell parameters from 9755 reflections
*b* = 8.6014 (19) Å	θ = 2.4–28.5°
*c* = 10.218 (2) Å	µ = 0.49 mm^−^^1^
α = 85.253 (10)°	*T* = 173 K
β = 81.478 (10)°	Block, colourless
γ = 83.961 (9)°	0.29 × 0.25 × 0.15 mm
*V* = 719.4 (3) Å^3^	

### Data collection

**Table:**

Bruker APEXII CCD diffractometer	3613 independent reflections
Radiation source: fine-focus sealed tube	3404 reflections with *I* > 2σ(*I*)
graphite	*R*_int_ = 0.025
φ and ω scans	θ_max_ = 28.5°, θ_min_ = 2.0°
Absorption correction: multi-scan (*SADABS*; Sheldrick, 1996)	*h* = −11→11
*T*_min_ = 0.871, *T*_max_ = 0.930	*k* = −11→11
13076 measured reflections	*l* = −13→13

### Refinement

**Table:**

Refinement on *F*^2^	Primary atom site location: structure-invariant direct methods
Least-squares matrix: full	Secondary atom site location: difference Fourier map
*R*[*F*^2^ > 2σ(*F*^2^)] = 0.031	Hydrogen site location: inferred from neighbouring sites
*wR*(*F*^2^) = 0.095	H-atom parameters constrained
*S* = 1.04	*w* = 1/[σ^2^(*F*_o_^2^) + (0.0575*P*)^2^ + 0.2633*P*] where *P* = (*F*_o_^2^ + 2*F*_c_^2^)/3
3613 reflections	(Δ/σ)_max_ < 0.001
174 parameters	Δρ_max_ = 0.34 e Å^−^^3^
0 restraints	Δρ_min_ = −0.44 e Å^−^^3^

### Special details

**Table:**

Geometry. All e.s.d.'s (except the e.s.d. in the dihedral angle between two l.s. planes) are estimated using the full covariance matrix. The cell e.s.d.'s are taken into account individually in the estimation of e.s.d.'s in distances, angles and torsion angles; correlations between e.s.d.'s in cell parameters are only used when they are defined by crystal symmetry. An approximate (isotropic) treatment of cell e.s.d.'s is used for estimating e.s.d.'s involving l.s. planes.
Refinement. Refinement of *F*^2^ against ALL reflections. The weighted *R*-factor *wR* and goodness of fit *S* are based on *F*^2^, conventional *R*-factors *R* are based on *F*, with *F* set to zero for negative *F*^2^. The threshold expression of *F*^2^ > σ(*F*^2^) is used only for calculating *R*-factors(gt) *etc*. and is not relevant to the choice of reflections for refinement. *R*-factors based on *F*^2^ are statistically about twice as large as those based on *F*, and *R*- factors based on ALL data will be even larger.

### Fractional atomic coordinates and isotropic or equivalent isotropic displacement parameters (Å^2^)

**Table:**

	*x*	*y*	*z*	*U*_iso_*/*U*_eq_	
S1	0.09888 (5)	0.15933 (5)	0.28102 (5)	0.03924 (12)	
S2	0.43827 (4)	0.15124 (4)	0.39691 (3)	0.02668 (10)	
P1	0.32713 (4)	0.16877 (4)	0.22735 (3)	0.02349 (10)	
O1	0.41933 (14)	0.04388 (11)	0.13285 (10)	0.0328 (2)	
O2	0.38169 (14)	0.31782 (11)	0.13866 (10)	0.0306 (2)	
O3	0.75419 (15)	0.31462 (15)	0.07640 (10)	0.0384 (3)	
O4	0.72161 (16)	0.33782 (15)	0.52567 (10)	0.0419 (3)	
N1	0.71376 (14)	0.29623 (13)	0.30631 (11)	0.0258 (2)	
C1	0.4028 (2)	−0.12177 (17)	0.16526 (19)	0.0450 (4)	
H1A	0.4367	−0.1523	0.2523	0.067*	
H1B	0.4716	−0.1830	0.0979	0.067*	
H1C	0.2889	−0.1418	0.1674	0.067*	
C2	0.3344 (2)	0.47159 (17)	0.18799 (18)	0.0412 (4)	
H2A	0.2154	0.4890	0.2049	0.062*	
H2B	0.3767	0.5518	0.1218	0.062*	
H2C	0.3791	0.4780	0.2706	0.062*	
C3	0.65366 (16)	0.14476 (16)	0.32840 (14)	0.0273 (3)	
H3A	0.7178	0.0791	0.3900	0.033*	
H3B	0.6704	0.0942	0.2432	0.033*	
C4	0.76056 (16)	0.36963 (16)	0.18021 (13)	0.0271 (3)	
C5	0.82125 (17)	0.51897 (16)	0.20613 (14)	0.0286 (3)	
C6	0.8801 (2)	0.63735 (19)	0.11828 (17)	0.0377 (3)	
H6	0.8815	0.6351	0.0253	0.045*	
C7	0.9376 (2)	0.76105 (19)	0.1734 (2)	0.0453 (4)	
H7	0.9779	0.8456	0.1165	0.054*	
C8	0.9370 (2)	0.76272 (19)	0.3089 (2)	0.0446 (4)	
H8	0.9788	0.8474	0.3428	0.053*	
C9	0.8763 (2)	0.64295 (19)	0.39698 (17)	0.0380 (3)	
H9	0.8758	0.6442	0.4899	0.046*	
C10	0.81732 (17)	0.52298 (17)	0.34284 (14)	0.0290 (3)	
C11	0.74723 (17)	0.38001 (17)	0.40935 (13)	0.0286 (3)	

### Atomic displacement parameters (Å^2^)

**Table:**

	*U*^11^	*U*^22^	*U*^33^	*U*^12^	*U*^13^	*U*^23^
S1	0.02281 (18)	0.0388 (2)	0.0582 (3)	−0.00383 (14)	−0.00798 (16)	−0.01070 (17)
S2	0.02212 (17)	0.03314 (18)	0.02380 (16)	−0.00471 (12)	−0.00268 (11)	0.00543 (12)
P1	0.02404 (18)	0.01980 (16)	0.02735 (17)	−0.00243 (12)	−0.00559 (12)	−0.00190 (12)
O1	0.0399 (6)	0.0230 (5)	0.0348 (5)	−0.0046 (4)	0.0011 (4)	−0.0068 (4)
O2	0.0425 (6)	0.0219 (4)	0.0278 (5)	−0.0033 (4)	−0.0083 (4)	0.0022 (3)
O3	0.0422 (6)	0.0503 (6)	0.0252 (5)	−0.0161 (5)	−0.0042 (4)	−0.0026 (4)
O4	0.0488 (7)	0.0529 (7)	0.0246 (5)	−0.0133 (5)	−0.0027 (4)	−0.0001 (5)
N1	0.0242 (5)	0.0295 (5)	0.0237 (5)	−0.0068 (4)	−0.0021 (4)	0.0008 (4)
C1	0.0568 (11)	0.0217 (6)	0.0540 (10)	−0.0065 (7)	0.0052 (8)	−0.0085 (6)
C2	0.0593 (11)	0.0198 (6)	0.0460 (9)	−0.0006 (6)	−0.0155 (7)	−0.0001 (6)
C3	0.0209 (6)	0.0269 (6)	0.0331 (7)	−0.0025 (5)	−0.0031 (5)	0.0027 (5)
C4	0.0217 (6)	0.0339 (6)	0.0255 (6)	−0.0062 (5)	−0.0031 (5)	0.0026 (5)
C5	0.0233 (6)	0.0297 (6)	0.0321 (7)	−0.0039 (5)	−0.0022 (5)	0.0014 (5)
C6	0.0334 (8)	0.0370 (7)	0.0407 (8)	−0.0067 (6)	−0.0020 (6)	0.0080 (6)
C7	0.0356 (8)	0.0301 (7)	0.0672 (11)	−0.0081 (6)	0.0004 (8)	0.0072 (7)
C8	0.0343 (8)	0.0304 (7)	0.0693 (12)	−0.0063 (6)	−0.0008 (7)	−0.0130 (7)
C9	0.0327 (8)	0.0365 (7)	0.0456 (8)	−0.0039 (6)	−0.0010 (6)	−0.0141 (6)
C10	0.0236 (6)	0.0296 (6)	0.0334 (7)	−0.0033 (5)	−0.0006 (5)	−0.0042 (5)
C11	0.0249 (6)	0.0336 (7)	0.0270 (6)	−0.0039 (5)	−0.0016 (5)	−0.0023 (5)

### Geometric parameters (Å, °)

**Table:**

S1—P1	1.9103 (6)	C2—H2B	0.9800
S2—C3	1.8261 (14)	C2—H2C	0.9800
S2—P1	2.0706 (6)	C3—H3A	0.9900
P1—O1	1.5671 (10)	C3—H3B	0.9900
P1—O2	1.5749 (10)	C4—C5	1.4864 (19)
O1—C1	1.4520 (18)	C5—C6	1.381 (2)
O2—C2	1.4494 (17)	C5—C10	1.396 (2)
O3—C4	1.2070 (18)	C6—C7	1.402 (2)
O4—C11	1.2081 (18)	C6—H6	0.9500
N1—C11	1.4003 (18)	C7—C8	1.386 (3)
N1—C4	1.4069 (17)	C7—H7	0.9500
N1—C3	1.4335 (17)	C8—C9	1.396 (2)
C1—H1A	0.9800	C8—H8	0.9500
C1—H1B	0.9800	C9—C10	1.377 (2)
C1—H1C	0.9800	C9—H9	0.9500
C2—H2A	0.9800	C10—C11	1.4870 (19)
			
C3—S2—P1	102.12 (5)	N1—C3—H3B	108.9
O1—P1—O2	96.75 (6)	S2—C3—H3B	108.9
O1—P1—S1	118.01 (5)	H3A—C3—H3B	107.7
O2—P1—S1	117.12 (5)	O3—C4—N1	124.76 (13)
O1—P1—S2	107.80 (5)	O3—C4—C5	129.98 (13)
O2—P1—S2	108.52 (4)	N1—C4—C5	105.23 (11)
S1—P1—S2	107.86 (3)	C6—C5—C10	121.80 (14)
C1—O1—P1	120.26 (10)	C6—C5—C4	129.95 (14)
C2—O2—P1	119.12 (10)	C10—C5—C4	108.20 (12)
C11—N1—C4	112.65 (11)	C5—C6—C7	116.55 (16)
C11—N1—C3	122.77 (11)	C5—C6—H6	121.7
C4—N1—C3	124.30 (12)	C7—C6—H6	121.7
O1—C1—H1A	109.5	C8—C7—C6	121.44 (15)
O1—C1—H1B	109.5	C8—C7—H7	119.3
H1A—C1—H1B	109.5	C6—C7—H7	119.3
O1—C1—H1C	109.5	C7—C8—C9	121.56 (15)
H1A—C1—H1C	109.5	C7—C8—H8	119.2
H1B—C1—H1C	109.5	C9—C8—H8	119.2
O2—C2—H2A	109.5	C10—C9—C8	116.89 (16)
O2—C2—H2B	109.5	C10—C9—H9	121.6
H2A—C2—H2B	109.5	C8—C9—H9	121.6
O2—C2—H2C	109.5	C9—C10—C5	121.73 (14)
H2A—C2—H2C	109.5	C9—C10—C11	129.60 (14)
H2B—C2—H2C	109.5	C5—C10—C11	108.65 (12)
N1—C3—S2	113.49 (9)	O4—C11—N1	124.50 (14)
N1—C3—H3A	108.9	O4—C11—C10	130.34 (14)
S2—C3—H3A	108.9	N1—C11—C10	105.15 (11)
			
C3—S2—P1—O1	46.79 (6)	C10—C5—C6—C7	0.8 (2)
C3—S2—P1—O2	−56.96 (6)	C4—C5—C6—C7	−176.42 (15)
C3—S2—P1—S1	175.23 (5)	C5—C6—C7—C8	0.8 (3)
O2—P1—O1—C1	−176.64 (13)	C6—C7—C8—C9	−1.2 (3)
S1—P1—O1—C1	−50.98 (14)	C7—C8—C9—C10	0.1 (2)
S2—P1—O1—C1	71.40 (13)	C8—C9—C10—C5	1.5 (2)
O1—P1—O2—C2	−176.16 (12)	C8—C9—C10—C11	179.35 (15)
S1—P1—O2—C2	57.54 (12)	C6—C5—C10—C9	−2.0 (2)
S2—P1—O2—C2	−64.80 (12)	C4—C5—C10—C9	175.78 (14)
C11—N1—C3—S2	75.68 (15)	C6—C5—C10—C11	179.78 (13)
C4—N1—C3—S2	−110.89 (13)	C4—C5—C10—C11	−2.49 (16)
P1—S2—C3—N1	90.77 (10)	C4—N1—C11—O4	−178.01 (14)
C11—N1—C4—O3	174.74 (14)	C3—N1—C11—O4	−3.9 (2)
C3—N1—C4—O3	0.7 (2)	C4—N1—C11—C10	1.89 (15)
C11—N1—C4—C5	−3.36 (15)	C3—N1—C11—C10	176.00 (12)
C3—N1—C4—C5	−177.37 (12)	C9—C10—C11—O4	2.3 (3)
O3—C4—C5—C6	3.1 (3)	C5—C10—C11—O4	−179.63 (16)
N1—C4—C5—C6	−178.98 (15)	C9—C10—C11—N1	−177.61 (15)
O3—C4—C5—C10	−174.42 (15)	C5—C10—C11—N1	0.48 (15)
N1—C4—C5—C10	3.54 (15)		

### Hydrogen-bond geometry (Å, °)

**Table:**

*D*—H···*A*	*D*—H	H···*A*	*D*···*A*	*D*—H···*A*
C2—H2B···O3^i^	0.98	2.57	3.272 (2)	128
C2—H2C···O4^ii^	0.98	2.70	3.420 (2)	130

Symmetry codes: (i) −*x*+1, −*y*+1, −*z*; (ii) −*x*+1, −*y*+1, −*z*+1.
